# Objectively measured habitual physical activity and sleep-related phenomena in 1645 people aged 1–91 years: The Nakanojo Community Study

**DOI:** 10.1016/j.pmedr.2018.06.013

**Published:** 2018-06-26

**Authors:** Yukitoshi Aoyagi, Sungjin Park, Sunyoung Cho, Roy J. Shephard

**Affiliations:** a Exercise Sciences Research Group, Tokyo Metropolitan Institute of Gerontology, Itabashi, Tokyo, Japan; b Faculty of Kinesiology and Physical Education, University of Toronto, Toronto, Ontario, Canada

**Keywords:** Axillary temperature, Chronic illness, Moderate-intensity exercise, Sleep efficiency, Step count

## Abstract

Relationships between habitual physical activity and sleep-related phenomena were examined in 623 male and 1022 female Japanese participating in the Nakanojo Community Study, using data collected in 2012–2013. Ages ranged from infancy to very old. Daily step count and daily duration of exercise at an intensity >3 metabolic equivalents (METs) were determined by pedometer/accelerometer, 24 h/day for 1 week. Duplicate axillary temperatures were also taken on rising and when retiring. Total bed time was noted, and the efficiency of sleep determined as hours of actual sleep (from a validated pedometer/accelerometer algorithm) divided by bed time. Step counts and especially duration of activity >3 METs peaked in teenagers and decreased as age advanced (p < 0.001). Both axillary temperatures subsequently showed a gradual age-related decline (p < 0.001). The duration and efficiency of sleep also showed a small age-dependent decrease (p < 0.001). Multivariate-adjusted correlation coefficients indicated a better quality of sleep in individuals who took greater habitual physical activity. In individuals aged ≥40 years, these findings were modified by chronic disease conditions including hypertension, diabetes mellitus and hyperlipemia; after controlling statistically for potential confounders, both physical activity and axillary temperature were lower (p < 0.05 or 0.01), and the time spent lying was longer but the efficiency of sleep was poorer (p < 0.01) in those with chronic conditions. These results suggest that habitual physical activity bears an important relationship to sleep-related phenomena at all ages, with a modification of relationships by chronic disease in people aged ≥40 years.

## Highlights

• Habitual physical activity is related to body temperature and sleep patterns.
• Body temperature is higher and sleep quality is better in physically active people.
• In people aged ≥40 years, these relationships are modified by chronic disease.
• Physical activity and body temperature are lower in older people with chronic disease.
• The time spent lying is longer but the quality of sleep is poorer in such people.

## 1 Introduction

The beneficial influence of habitual physical activity upon many chronic diseases is well-documented for people from infancy to an advanced age (Bouchard et al., 1994; Garber et al., 2011; Shephard, 1997a, Shephard, 1997b; Sothern et al., 1999). Published articles have investigated relationships between physical activity and sleep patterns (see Kredlow et al., 2015 for review), but possible changes in these relationships at various points in life and the modifying effects of chronic disease are much less clearly understood. We thus decided to explore these issues, exploiting extensive data collected as a part of the Nakanojo Study (Aoyagi and Shephard, 2009, Aoyagi and Shephard, 2010, Aoyagi and Shephard, 2011, Aoyagi and Shephard, 2013, Aoyagi and Shephard, 2014).

Age is closely associated with an individual's level of habitual physical activity, average body temperatures, the quality of sleep and the incidence of lifestyle-related diseases (Shephard, 1997a). With aging, most people show (i) a progressive decrease in both the quantity and the quality of habitual physical activity (Aoyagi and Shephard, 1992, Aoyagi and Shephard, 2009; Yasunaga et al., 2008); (ii) changes in body temperature including a reduction in mean values, a narrowing of diurnal variations and a phase advance of peak values (Czeisler et al., 1992; Duffy et al., 1998); (iii) a reduction in measures of sleep efficiency such as increases in the time required to fall asleep (sleep latency) and reach the phase of deep sleep (deep sleep latency), and a decrease in the ratio of stage 3 and 4 deep sleep to the total sleep per night (Czeisler et al., 1992; Duffy et al., 1998); and (iv) an increase in the prevalence of chronic lifestyle-related conditions such as hypertension, type 2 diabetes mellitus and hyperlipemia (Ministry of Health, Labour and Welfare, 2017).

Pedometry/accelerometry studies have already clarified some of the thresholds of habitual physical activity associated with health markers in specific age groups. For example, Aoyagi and Shephard, 2009, Aoyagi and Shephard, 2010, Aoyagi and Shephard, 2011, Aoyagi and Shephard, 2013, Aoyagi and Shephard, 2014 have shown that after adjustment for potential confounders, the overall health of individuals aged ≥65 years is associated with both the year-averaged daily step count (the best indicator in women) and the year-averaged daily duration of physical activity at an intensity >3 metabolic equivalents (METs) (the best indicator in men). In ostensibly healthy people of both sexes aged ≥65 years, these thresholds are: (i) 4000–5000 steps/day and/or 5–7.5 min/day for impaired mental and psychosocial health, such as a depressed mood state (Yoshiuchi et al., 2006) and a poor health-related quality of life (Yasunaga et al., 2006); (ii) 7000–8000 steps/day and/or 15–20 min/day for markers of aortic arteriosclerosis (Aoyagi et al., 2010), osteoporosis (Park et al., 2007; Shephard et al., 2017), sarcopenia (Park et al., 2010; Shephard et al., 2013) and poor physical fitness (Aoyagi et al., 2009); and (iii) 8000–10,000 steps/day and/or 20–30 min/day for components of the metabolic syndrome, especially hypertension and hyperglycemia (Park et al., 2008).

Body temperature has been considered a “gold-standard” marker of human circadian rhythm, and it is strongly related to sleep regulation (Togo et al., 2007). The human sleep/wake cycle is generally synchronized with the circadian rhythm of body temperature, partly because the propensity for sleep reaches its maximum during the declining phase of body temperature and partly because arousal is promoted after body temperature has passed its nadir (Borbély, 1982; Dijk and Czeisler, 1995). Body temperature decreases rapidly about 60 min prior to the onset of sleep (Murphy and Campbell, 1997), and it decreases further during stage 3 and 4 deep sleep, due to a combination of decreases in metabolism, vasodilation and an increase in sweating (Glotzbach and Heller, 1976; Sagot et al., 1987). Deep sleep is related to brain or whole-body cooling, and such heat loss is thought to be associated with increases in brain and body temperatures during the preceding period of wakefulness. If brain and body temperatures are increased by either exercise or passive heating while a person is awake, then the amount of deep sleep is increased during the ensuing night (Horne and Reid, 1985; Horne and Staff, 1983). The amount of deep sleep is positively correlated with both body temperature at sleep onset (Berger et al., 1988) and the magnitude of the decrease in body temperature during sleep (Horne and Staff, 1983), so that even quite small changes in body temperature may influence sleep patterns.

However, the normal relationship of habitual physical activity to body temperature and sleep-related phenomena, and the impact of lifestyle-related diseases upon this relationship at various points in the life cycle remain unclear. The present study thus investigated these relationships in a large population over a wide age range. Our hypotheses were (i) that habitual physical activity, body temperature and sleep state would each decrease with aging in parallel but different manners; (ii) that greater habitual physical activity would be associated with higher evening body temperatures and thus better sleep patterns; and (iii) that age- and chronic disease-related decreases in habitual physical activity would be marked by lower body temperatures and impairments of sleep.

## 2 Methods

### 2.1 Subjects

The subjects were 623 male and 1022 female free-living volunteers from the community of Nakanojo Town, Gunma Prefecture, Japan. The age distribution included 148 younger children (0–9 years), 212 older children (10–19 years), 316 younger adults (20–39 years), 621 older adults (40–64 years) and 348 seniors (65–<100 years). Criteria of recruitment included willingness to participate, attendance at an annual medical examination, functional independence (for the elderly), and the absence of chronic or progressive conditions that could limit physical activity or have a major effect on the individual's perceived quality of life (e.g., cancer, arthritic diseases, Parkinson's disease, Alzheimer's disease, multiple sclerosis, amyotrophic lateral sclerosis, and dementia). In the period 2012–2013, subjects (and the parents or guardians of minors) gave their written informed consent to participation in a study approved by the ethics review committee of the Tokyo Metropolitan Institute of Gerontology, after the protocol, stresses and possible risks had been fully explained to them. Table 1 summarizes the physical characteristics of the subjects.

**Table 1:** Selected anthropometric, physical activity, axillary temperature and sleep state measurements of subject groups.

	All (n = 1645)	Men (n = 623)	Women (n = 1022)
Age (years)	42.9 ± 23.2	42.0 ± 24.1	43.5 ± 22.6
Height (m)	1.54 ± 0.20	1.59 ± 0.22	1.50 ± 0.17***
Body mass (kg)	52.9 ± 16.0	58.8 ± 18.6	49.2 ± 12.9***
Body mass index (kg/m^2^)	21.7 ± 3.7	22.2 ± 4.0	21.4 ± 3.5***
Week-averaged step count (steps/day)	6445 ± 2840	6951 ± 3092	6149 ± 2640***
Week-averaged duration of physical activity >3 METs (min/day)	17.1 ± 12.5	19.5 ± 13.8	15.6 ± 11.4***
Week-averaged hour of rising	6:15 ± 0:48	6:20 ± 0:50	6:12 ± 0:47**
Week-averaged hour of going to bed	22:51 ± 1:30	22:51 ± 2:02	22:51 ± 1:05
Week-averaged axillary temperature on rising (°C)	36.25 ± 0.26	36.24 ± 0.25	36.25 ± 0.27
Week-averaged axillary temperature on going to bed (°C)	36.21 ± 0.33	36.25 ± 0.32	36.18 ± 0.33***
Week-averaged sleep duration (h/night)	5:52 ± 1:20	5:58 ± 1:31	5:49 ± 1:13*
Week-averaged sleep efficiency (%)	78.8 ± 12.8	78.5 ± 14.5	78.9 ± 11.6

Values are mean ± SD.

METs = metabolic equivalents.

*, ** and ***p < 0.05, p < 0.01 and p < 0.001, respectively, versus men.

### 2.2 Physical activity measurement

Physical activity patterns were measured for 24 h per day over a 1-week period, using a uniaxial acceleration sensor (Lifecorder; Suzuken Co. Ltd., Nagoya, Aichi, Japan), as described previously (Aoyagi and Shephard, 2009, Aoyagi and Shephard, 2010, Aoyagi and Shephard, 2011, Aoyagi and Shephard, 2013, Aoyagi and Shephard, 2014). The 1 week of observations excludes the potential effects of day of the week, and although vulnerable to reactive and seasonal effects, it provides sufficient data to predict yearlong physical activity with >80% reliability at least in older people with a relatively regular daily pattern (Togo et al., 2008). The average number of steps taken per day and the daily cumulative duration of moderate-intensity physical activity (at an intensity >3 metabolic equivalents [METs]; Garber et al., 2011) were calculated for each subject.

### 2.3 Axillary temperature measurement

Axillary temperatures were measured over the same 1-week period as physical activity, after subjects had lain in bed quietly for a few minutes. All measurements were made using a clinical thermometer (ET-C231P; Terumo Corporation, Shibuya, Tokyo, Japan). Two observations were made, both on rising and on going to bed; the average of the two readings was recorded.

### 2.4 Sleep state measurements

Total sleep time was calculated from bedtime to the hour of rising, as recorded by the subjects (or the parents in the case of the children) over the same 1-week period as for measurements of physical activity and axillary temperature. Hours of actual sleep were determined by a validated pedometer/accelerometer algorithm (SleepSignAct; Kissei Comtec Co. Ltd., Matsumoto, Nagano, Japan); this has a mean agreement rate with the corresponding polysomnography-based sleep/wake data of 86.9% (Enomoto et al., 2009). The efficiency of sleep was estimated as the hours of actual sleep divided by the time spent lying in bed.

### 2.5 Lifestyle-related diseases

Lifestyle-related diseases that were included in the present analysis comprised hypertension, type 2 diabetes mellitus and dyslipidemia (hyperlipidemia). Diagnoses were based on a physician-elicited self-report and/or the Nakanojo Study (Aoyagi and Shephard, 2009, Aoyagi and Shephard, 2010, Aoyagi and Shephard, 2011, Aoyagi and Shephard, 2013, Aoyagi and Shephard, 2014). Subjects were diagnosed as having (i) hypertension, (ii) diabetes mellitus and/or (iii) hyperlipidemia if the following criteria were met: (i) systolic pressure ≥140 mm Hg and/or diastolic pressure ≥90 mm Hg; (ii) a fasting plasma glucose ≥126 mg/dL and/or a hemoglobin A1c ≥6.5% and/or (iii) a fasting serum low-density lipoprotein cholesterol concentration ≥140 mg/dL, a fasting serum high-density lipoprotein cholesterol concentration <40 mg/dL and/or a fasting serum triglyceride concentration ≥150 mg/dL; and/or a doctor's pronouncement and/or the currently taking of prescribed (i) antihypertensive, (ii) hypoglycemic and/or (iii) hypocholesterolemic medications.

### 2.6 Statistical analyses

The SPSS 23.0 (IBM Corp., Armonk, NY, USA) was used throughout. Non-paired *t*-tests analyzed sex differences in anthropometric characteristics, physical activity patterns, hours of rising and going to bed, axillary temperatures and sleep states. Partial correlation analyses assessed independent associations (i) between age and each of physical activity, axillary temperature or sleep state in all subjects, after controlling as appropriate for sex, body mass index, smoking status, alcohol consumption, employment status and/or hypnotic use; and (ii) between physical activity, axillary temperature and sleep state parameters in all subjects, after controlling as appropriate for age, sex, body mass index, smoking status, alcohol consumption, employment status and/or hypnotic use. Subjects were divided into nine arbitrary groups on the basis of age, or (if their ages were ≥40 years) two arbitrary groups on the basis of the presence/absence of one or more of lifestyle-related diseases. Analysis of covariance (ANOCOVA) assessed independent differences between these nine or two groups with respect to physical activity, axillary temperature and sleep state variables, after controlling as appropriate for age, sex, body mass index, smoking status, alcohol consumption, employment status and/or hypnotic use. A Dunnett correction was applied when multiple comparisons were made between the youngest group and the others. The ANOCOVA also assessed differences between the two variables of axillary temperature (on rising versus on going to bed) within each age group, after adjustment as appropriate for sex, body mass index, smoking status, alcohol consumption, employment status and/or hypnotic use. All statistical contrasts were made at the 0.05 level of significance.

## 3 Results

### 3.1 Subject characteristics

We observed the anticipated differences of height, body mass and body mass index between male and female subjects (all p < 0.001), but the mean age did not differ significantly with sex (Table 1). Habitual physical activity was greater in males than in females, significantly so (p < 0.001) for both the week-averaged daily step count and the week-averaged daily duration of activity at an intensity >3 METs. Similarly, the hour of rising was significantly later (p < 0.01), the axillary temperature when going to bed was higher (p < 0.001) and the sleep duration longer (p < 0.05) in males than in females. However, there were no statistically significant differences in the hour of going to bed, axillary temperature when rising or sleep efficiency between sexes.

### 3.2 Age-related changes in physical activity

**Fig. 1:**
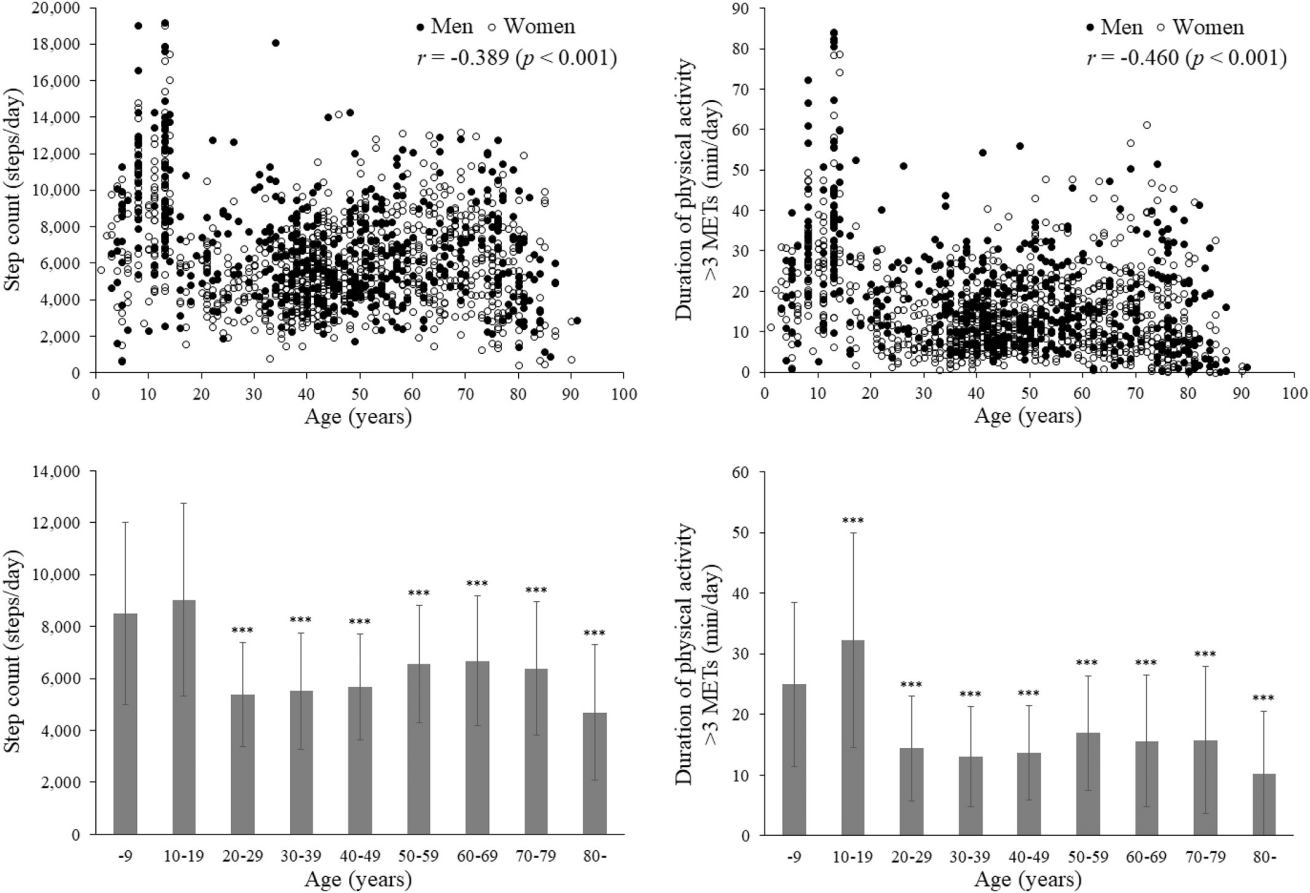
The week-averaged daily step count (left) and daily duration of physical activity at an intensity >3 metabolic equivalents (METs) (right), plotted against individual values (upper) and classified by groups in 10-year increments of age (lower). n = 1449. In the lower figures, values are mean ± SD. ***p < 0.001 versus the group of subjects aged ≤9 years after adjustment for potential confounders.

The week-averaged daily step count and daily duration of physical activity at an intensity >3 METs peaked in teenagers (significantly higher for the latter relative to a reference group of subjects aged ≤9 years drawn from the same population; p < 0.001), and decreased significantly (p < 0.001) at greater ages (Fig. 1), differences being larger for the duration of activity >3 METs (r = −0.460) than for the step count (r = −0.389). Inter-individual variations in physical activity patterns were greater in younger subjects (especially aged ≤19 years), but all subjects aged ≥20 years were significantly (p < 0.001) less active than those aged ≤19 years in terms of both step count and duration of activity >3 METs.

### 3.3 Age-related changes in axillary temperature

**Fig. 2:**
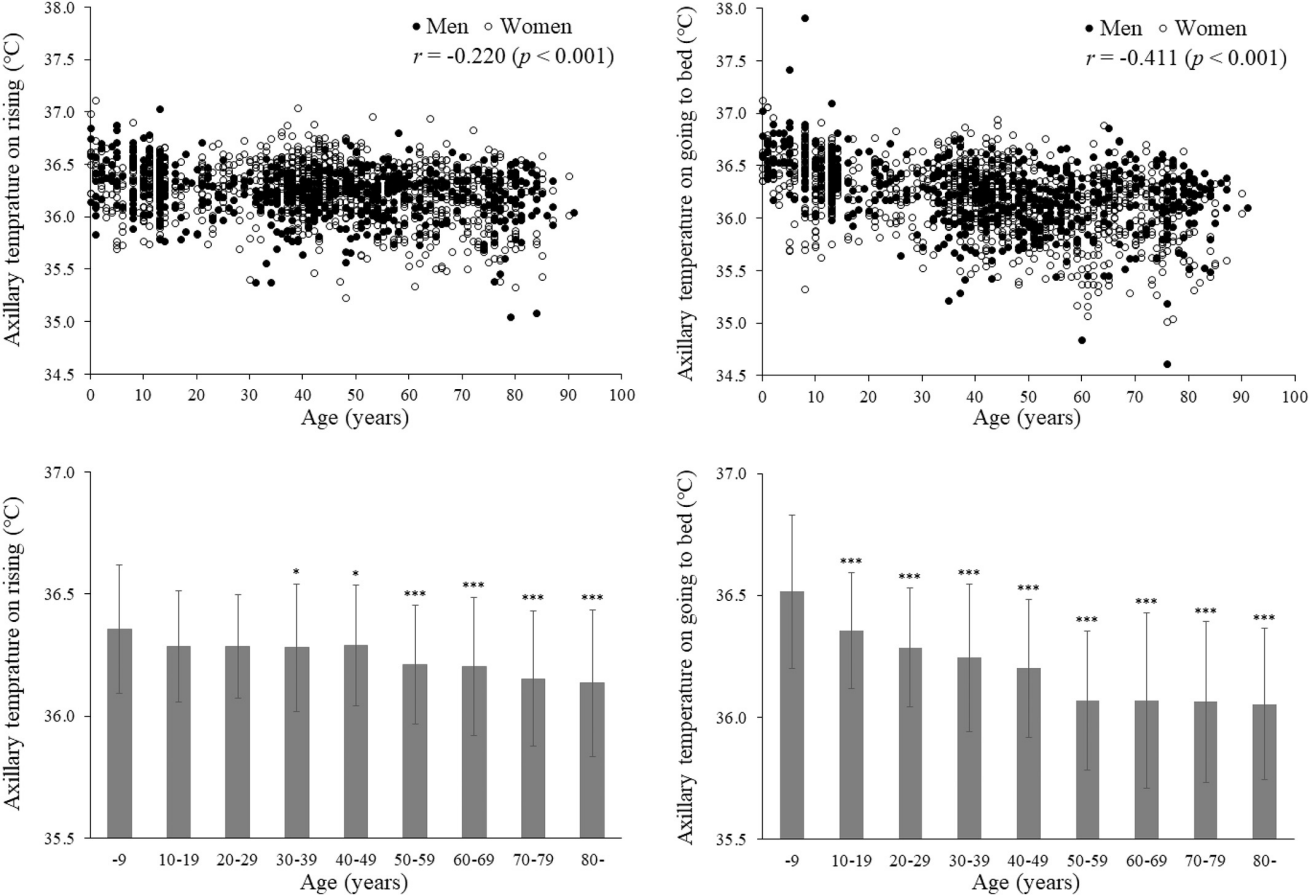
The week-averaged axillary temperatures when rising (left) and when going to bed (right), plotted against individual values (upper) and classified by groups in 10-year increments of age (lower). n = 1645. In the lower figures, values are mean ± SD. * and ***p < 0.05 and p < 0.001, respectively, versus the group of subjects aged ≤9 years after adjustment for potential confounders.

Week-averaged axillary temperatures were significantly (p < 0.001) lower in older individuals (Fig. 2), both when rising (r = −0.220) and when going to bed (r = −0.411). Differences between axillary temperatures when rising and when going to bed were statistically significant (p < 0.05, 0.01 or 0.001) in all age groups except those in their twenties and thirties.

### 3.4 Age-related changes in sleep state

**Fig. 3:**
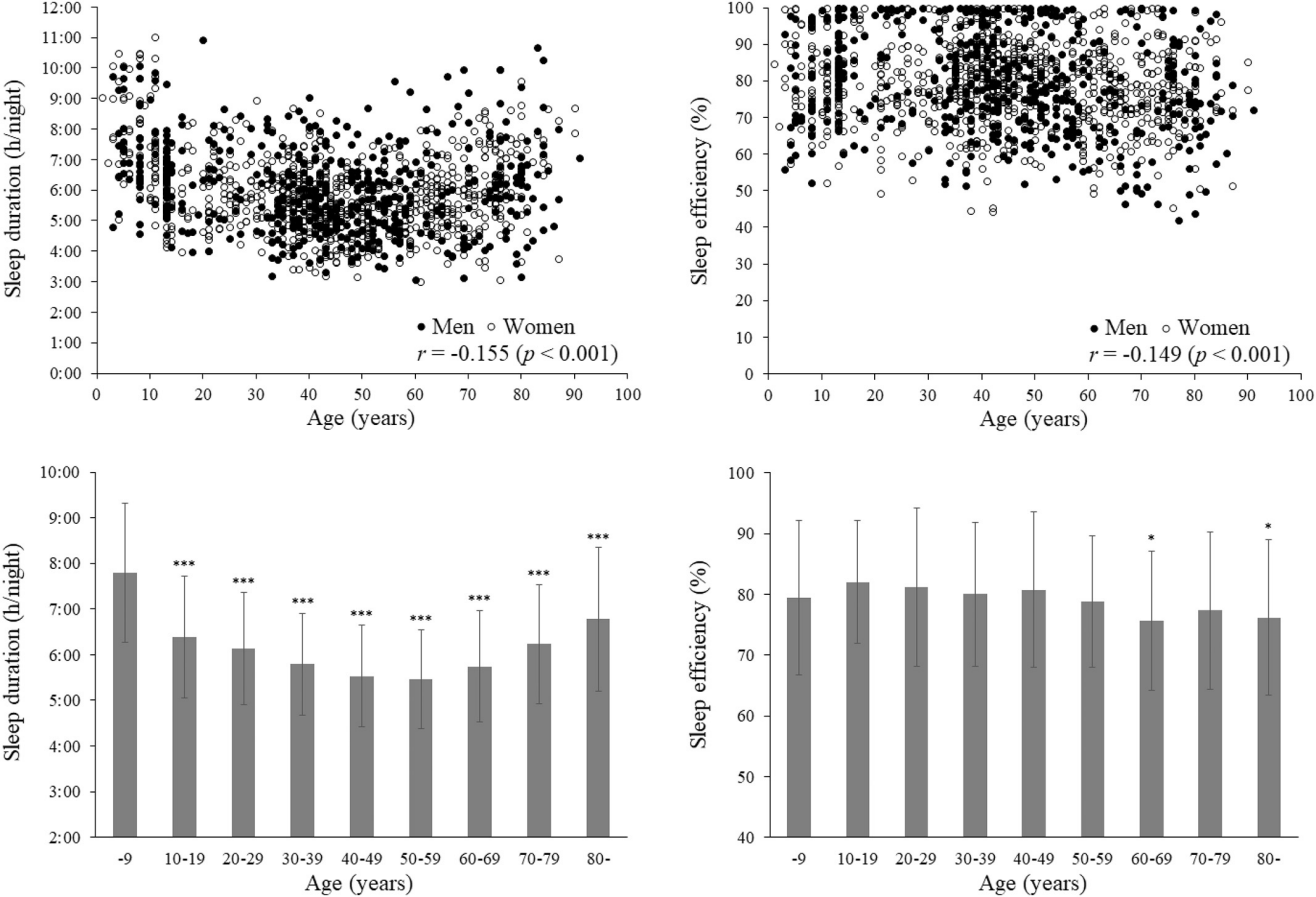
The week-averaged sleep duration (left) and sleep efficiency (right), plotted against individual values (upper) and classified by groups in 10-year increments of age (lower). n = 1424. In the lower figures, values are mean ± SD. * and ***p < 0.05 and p < 0.001, respectively, versus the group of subjects aged ≤9 years after adjustment for potential confounders.

Both duration and efficiency of sleep showed a small but statistically significant (p < 0.001) age-dependent decrease (r = −0.155 and −0.149, respectively; Fig. 3). Relative to a reference group of subjects aged ≤9 years drawn from the same population, all other age groups had shorter sleep durations (p < 0.001), and the two groups of subjects in their sixties and aged ≥80 years also had lower sleep efficiencies (p < 0.05).

### 3.5 Interrelationships between physical activity, axillary temperature and sleep state

Multivariate-adjusted correlation coefficients (Table 2) indicated higher axillary temperatures (particularly when going to bed) and better sleep states in the more active individuals (particularly in terms of the daily duration of activity at an intensity >3 METs). Statistically significant correlations were found between the two physical activity variables (r = 0.867; p < 0.001), axillary temperatures when rising and retiring (r = 0.477; p < 0.001), and sleep duration versus efficiency of sleep (r = 0.674; p < 0.001), with smaller correlations between step count and axillary temperatures both when rising (r = 0.059; p < 0.05) and when going to bed (r = 0.136; p < 0.01), between duration of activity >3 METs and axillary temperature when going to bed (r = 0.125; p < 0.01), duration and efficiency of sleep (r = 0.092 and 0.087, respectively; p < 0.01), and between axillary temperature when going to bed and sleep duration (r = 0.125; p < 0.01) and efficiency (r = 0.059; p < 0.05).

**Table 2:** Partial correlation coefficients between selected parameters of physical activity, axillary temperature and sleep state in subjects (n = 1415).

	Step count	Duration of physical activity >3 METs	Axillary temperature on rising	Axillary temperature on going to bed	Sleep duration	Sleep efficiency
Step count	1	0.867***	0.059*	0.136**	0.049	0.026
Duration of physical activity >3 METs	–	1	0.029	0.125**	0.092**	0.087**
Axillary temperature on rising	–	–	1	0.477***	0.032	0.042
Axillary temperature on going to bed	–	–	–	1	0.125**	0.059*
Sleep duration	–	–	–	–	1	0.674***
Sleep efficiency	–	–	–	–	–	1

METs = metabolic equivalents.

*, ** and ***p < 0.05, p < 0.01 and p < 0.001, respectively, after adjustment for potential confounders.

### 3.6 Physical activity, axillary temperature and sleep state in those with chronic diseases

After controlling as appropriate for potential confounders, there were statistically significant (p < 0.05 or 0.01) differences in all variables except sleep duration between subjects aged ≥40 years with lifestyle-related diseases (including hypertension, diabetes mellitus and hyperlipemia) and those free of such conditions (Table 3). Physical activity (both step count and duration of activity >3 METs) and axillary temperatures (both on rising and on going to bed) were significantly lower, and the time spent lying in bed was longer but the efficiency of sleep was poorer in individuals aged ≥40 years with hypertension, diabetes mellitus and/or hyperlipemia (than in those without such diseases).

**Table 3:** Selected parameters of physical activity, axillary temperature and sleep state in individuals aged ≥40 years with versus without lifestyle-related diseases (including hypertension, diabetes mellitus and hyperlipemia).

	With diseases (n = 217)	Without diseases (n = 453)
Step count (steps/day)	5707 ± 2287	6160 ± 2209*
Duration of physical activity >3 METs (min/day)	12.6 ± 9.3	15.3 ± 9.5**
Axillary temperature on rising (°C)	36.14 ± 0.25	36.28 ± 0.25**
Axillary temperature on going to bed (°C)	36.05 ± 0.32	36.15 ± 0.30*
Sleep duration (h/night)	5:39 ± 1:25	5:36 ± 1:13
Sleep efficiency (%)	74.3 ± 13.4	79.4 ± 12.0**

Values are mean ± SD.

METs = metabolic equivalents.

* and **p < 0.05 and p < 0.01, respectively, versus individuals with diseases after adjustment for potential confounders.

## 4 Discussion

In keeping with our initial hypotheses, this cross-sectional epidemiological study supports previous experimental and clinical observations (Berger et al., 1988; Horne and Staff, 1983; Togo et al., 2007) in showing associations among physical activity, body temperature, sleep and lifestyle-related diseases in a Japanese population. The new epidemiological data demonstrate that after adjusting data for potential confounders, (i) the variables of habitual physical activity (particularly daily duration of activity >3 METs), axillary temperature (particularly when going to bed) and sleep state decrease in older individuals in parallel but somewhat different manners; (ii) axillary temperatures (particularly when going to bed) are higher and sleep states better in physically more active individuals (particularly in terms of duration at >3 METs); and (iii) in individuals aged ≥40 years with hypertension, diabetes mellitus and/or hyperlipemia, physical activity (both step count and duration at >3 METs) and axillary temperatures (both on rising and on going to bed) are lower, and the time spent lying in bed is longer but the efficiency of sleep is poorer relative to those who do not have such conditions. Although the multivariate-adjusted differences and relationships between the variables are relatively small, they may nevertheless have physiological significance.

Even small differences in evening axillary temperature could influence sleep latency. Axillary temperatures when going to bed showed a greater age-related decline than those seen when rising, and the temperature on retiring became notably lower than on rising in those over the age of 40 years. This might reflect in part earlier times of retiring in people aged ≥40 years, but it probably reflects more an increased prevalence of chronic disease (Ministry of Health, Labour and Welfare, 2017) and an age- and disease-related decrease in habitual physical activity. The progressive decrease of habitual physical activity with age has been documented previously (Aoyagi and Shephard, 1992, Aoyagi and Shephard, 2009; Yasunaga et al., 2008). The present cross-sectional study confirms this trend over a wide age range, showing inverse associations between age and both the week-averaged daily step count and the week-averaged daily duration of physical activity at an intensity >3 METs. Further, the age-related changes in physical activity were greater for duration of activity >3 METs than for step count. Nevertheless, such changes were less clear than those in axillary temperature, mainly because of the large inter-individual differences in physical activity among younger subjects (especially those aged ≤19 years) and the lesser volumes of physical activity seen in adults (especially among those in their twenties to forties). At least three factors contribute to these discrepancies: (i) there may be cohort effects from secular changes of lifestyle; (ii) regardless of age, many adults use their own automobiles for transportation, particularly in rural Japan; and (iii) many traditional Japanese women (most of our sample would fit this characterization) spend long periods performing household tasks. In addition, most younger female adults are heavily involved with both housework and child rearing.

Our previous studies (Aoyagi and Shephard, 2009, Aoyagi and Shephard, 2010, Aoyagi and Shephard, 2011, Aoyagi and Shephard, 2013, Aoyagi and Shephard, 2014) have shown that after adjustment for potential confounders, the mental, psychosocial, physical and metabolic health of older people is associated with both the quantity (daily step count) and the quality (daily duration at an intensity >3 METs) of habitual physical activity. Many other reports (e.g., Aoyagi and Shephard, 1992; Shephard, 1997a, Shephard, 1997b) have indicated that regular physical activity induces an increase of muscle mass and of immune function, irrespective of age or sex. The detailed mechanisms of interactions between physical activity, axillary temperature and sleep patterns, and their modification by chronic disease remain unclear. In particular, the evening axillary temperatures might reflect either the influence of recent physical activity or the impact of a greater muscle mass and thus a higher resting metabolic rate. Possibly, persons in middle and old age who maintain a physically active lifestyle slow the typical age-dependent reduction in muscle mass, thus maintaining higher levels of basic metabolism and body temperature, with a higher evening body temperature facilitating better sleep patterns.

There are some limitations to the present investigation. Because of the number of subjects involved, it was necessary to shorten the period for the measurement of physical activity and other variables to the 7 days adopted by most epidemiologists, leaving the data somewhat vulnerable to both reactive effects and non-representative sampling of activity patterns (although individual subjects were studied at differing points over the year to avoid issues of seasonal change). The age range was also wide, so that the criterion of moderate physical activity (a standard intensity >3 METs, as adopted in our earlier studies) was more taxing for the elderly (aged ≥65 years) than for younger (≤64-year-old) individuals (Garber et al., 2011). The design was cross-sectional rather than longitudinal, so that causation cannot be inferred. The most common means of transport also differs from one region to another; in the Nakanojo area (a rural Japanese town), younger adults usually travel by private automobile (or rarely by public transportation, as is more common in urban and suburban environments) rather than on foot or by bicycle; however, walking was common for elderly women and very old men in Nakanojo. There remains a need to explore whether findings would be similar in other communities with environments more favorable to active transportation over substantial distances. On the other hand, an important strength of this investigation is that measurements of physical activity, axillary temperature and sleep patterns were made objectively by devices such as the pedometer/accelerometer and a clinical thermometer. Furthermore, subjects were evaluated regularly for the possible onset of hypertension, diabetes mellitus and hyperlipemia, using a standardized methodology, as a part of the ongoing Nakanojo Study (Aoyagi and Shephard, 2009, Aoyagi and Shephard, 2010, Aoyagi and Shephard, 2011, Aoyagi and Shephard, 2013, Aoyagi and Shephard, 2014). These aspects of the investigation strengthen its practical significance in terms of the observed relationships between habitual physical activity, axillary temperature and sleep patterns, and their modifications by the development of lifestyle-related diseases.

## 5 Conclusions

After adjustment for potential confounders, habitual physical activity is related to body temperature and sleep patterns, with disturbance of these relationships by the onset of lifestyle-related diseases. Axillary temperatures (particularly when going to bed) are higher and better-quality sleep is seen in those who are physically more active (particularly in terms of daily duration of physical activity at an intensity >3 METs). In individuals aged ≥40 years with hypertension, diabetes mellitus and/or hyperlipemia, both habitual physical activity (step count and duration of activity >3 METs) and axillary temperatures (on rising and on going to bed) are lower, and the time spent lying in bed is longer but the efficiency of sleep is poorer relative to those who remain healthy. Nevertheless, interventional studies are needed to determine whether the observed associations reflect a causal influence of habitual physical activity upon sleep-related phenomena or vice versa.

## Conflicts of interest

None declared.

## Funding

This study was supported in part by grants (Grant-in-Aid for Encouragement of Young Scientists: 12770037 and Grant-in-Aid for Scientific Research [C]: 15500503, [C]: 17500493, [B]: 19300235, and [B]: 23300259) from the Japan Society for the Promotion of Science and a grant from the Terumo Corporation.
